# Supplementary material for: The interactome of intact mitochondria by cross-linking mass spectrometry provides evidence for coexisting respiratory supercomplexes
Source: Mol Cell Proteomics. 2017 Dec 8;17(2):216–32. doi: 10.1074/mcp.RA117.000470 (PMC5795388; doi:10.1074/mcp.RA117.000470)
Supplement: Supplemental Data [file supp_RA117.000470_133922_0_supp_23978_fzffwf.zip › spectra_annotation/mito_DR_spectra_annotation/260-1-1-1-49-1.pdf]

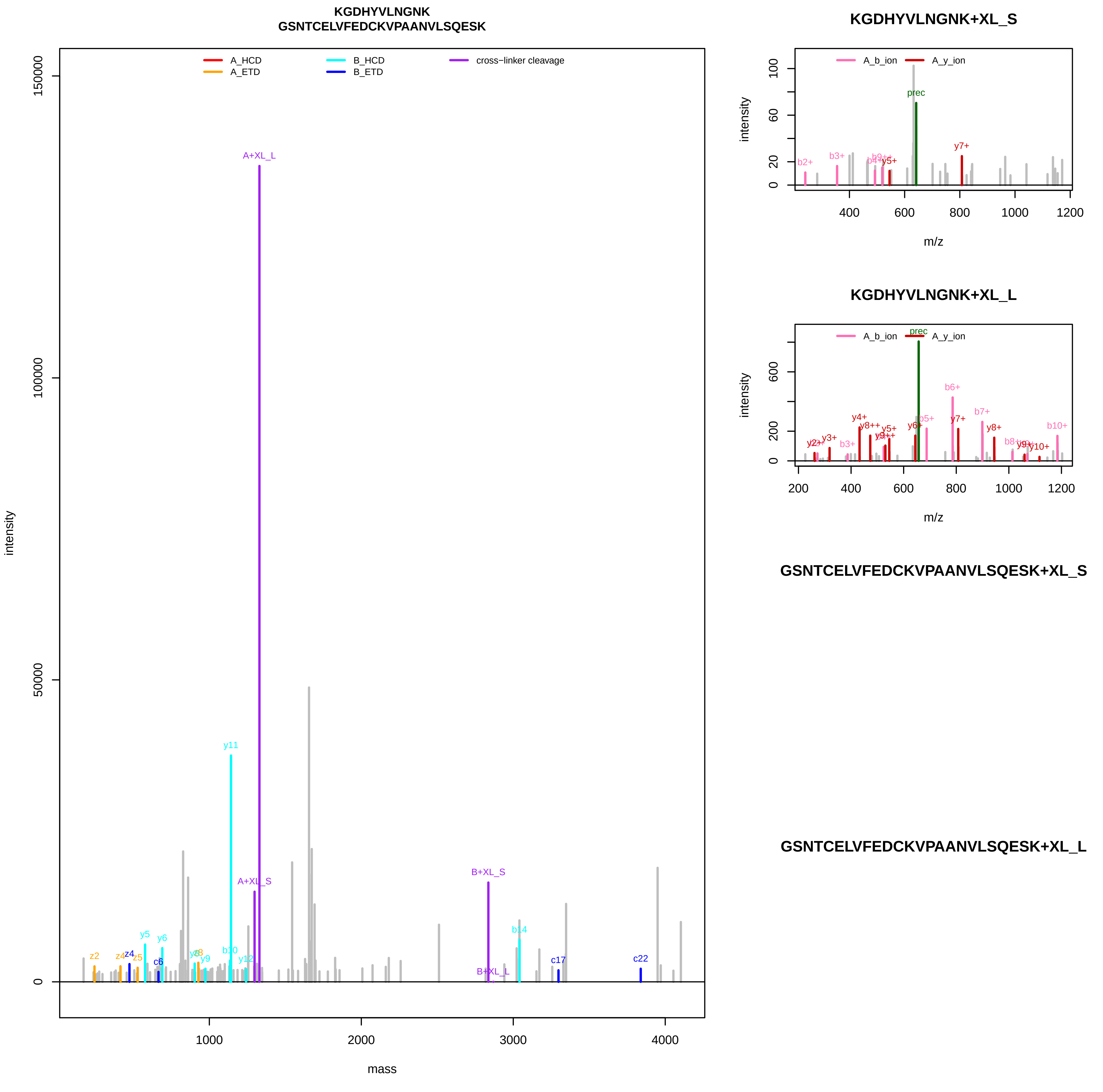
KGDHYVLNGNK
GSNTCELVFEDCKVPAANVLSQESK
A_HCD
A_ETD
B_HCD
B_ETD
cross−linker cleavage
A+XL_L
A+XL_S
B+XL_S
B+XL_L
y11
y5
y6
b10
y12
b14
c6
c17
c22
z2
z4
z5
y9
intensity
mass
KGDHYVLNGNK+XL_S
A_b_ion
A_y_ion
prec
y7+
b2+
b3+
y5+
m/z
KGDHYVLNGNK+XL_L
b6+
b7+
b10+
y4+
y8++
y3+
y2+
b3+
y7+
y8+
y6+
b5+
y10+
GSNTCELVFEDCKVPAANVLSQESK+XL_S
GSNTCELVFEDCKVPAANVLSQESK+XL_L
